# Diagnosing structural narrowing in public digital health information on healthy eating: a stratified search-entry analysis of Xiaohongshu posts

**DOI:** 10.3389/fpubh.2026.1977016

**Published:** 2026-10-20

**Authors:** Qingyong Xu, Tong Qi, Hangyu Zhou, Chunchun Sun

**Affiliations:** 1 Law School, Qingdao University of Science and Technology, Qingdao, Shandong, China; 2 School of Law, Qingdao University, Qingdao, Shandong, China

**Keywords:** content analysis, digital health governance, digital health information, healthy eating, information narrowing, lifestyle management, social media, user-generated content

## Abstract

**Objective:**

User-generated platforms now function as everyday sources of dietary guidance, making their visible content part of the public digital health information environment. This study diagnoses how healthy-eating posts on Xiaohongshu are organized across health goals, food or method pathways, and applicability conditions, and compares these structures across search entries and levels of public interaction. Structural narrowing is treated as a multidimensional pattern rather than as simple concentration around a search topic.

**Methods:**

We manually accessed publicly searchable image-text posts through the Xiaohongshu mobile application between 28 July 2026 at 17:56:43 and 30 July 2026 at 13:25:47. Six search-entry categories each contained four original Chinese keywords, and 25 qualifying posts were sampled per keyword, producing 600 search-occurrence records. Duplicate views within a keyword were excluded during collection. When the same post appeared under more than one keyword, its search occurrences were retained for entry-level comparison but collapsed into one record when the unique-post corpus was formed. After manual review and deduplication, 594 unique core posts remained. Content was coded for health goals, food and method pathways, templated presentation, advice or effect claims, and condition disclosure. Overall structure and interaction patterns were analyzed using unique posts, while entry differences were examined using occurrence records. Two paired coding rounds were used to calculate Cohen’s kappa for the core fields.

**Results:**

The 594 unique core posts were concentrated around weight/body-shape goals and restriction/control pathways, although the range of food objects was not uniformly narrow. Different search entries showed different combinations of structural features. Weight/body-shape entries emphasized weight goals and restriction/control; bodily-indicator and disease-state entries combined state management with control; method entries organized eating around rhythm-based schemes and templates; product entries shifted attention toward productized objects and weaker condition disclosure; and templated entries foregrounded replicable arrangements. Highly interactive posts were more concentrated in weight, control, and templated structures, but not in insufficient condition disclosure. Among posts that made advice or effect claims, some lacked information on target population, dosage, frequency, duration, or risks.

**Conclusion:**

On Xiaohongshu, narrowing was expressed less through the disappearance of food diversity than through repeated combinations of goals, methods, presentation formats, and applicability conditions. A keyword’s topical focus alone was not treated as narrowing. The diagnosis required multiple features, such as concentrated goals, compressed methods, object substitution, templating, or weakened condition boundaries, to occur together within an entry. The findings support entry-level monitoring of public digital health information while remaining distinct from clinical safety assessment, patient-level research, and claims about user adoption.

## 1 Introduction

In the public digital health information environment, healthy eating has moved beyond professional guidelines and institutional education into ordinary lifestyle management. Users now encounter dietary information on social platforms through practical formats such as fat-loss meals, glucose-control recipes, intermittent-fasting plans, meal-replacement recommendations, and weekly menus. Influencer content and food displays reshape the platform food environment (1, 2), social media is used as a nutrition information source by some younger groups (3, 4), and digital food communication can influence how young users understand and respond to food and nutrition (5). Against a policy background in which prevention, lifestyle management, medical security, and health-data governance are increasingly discussed together, platform-based dietary content is more than personal sharing. It is also an upstream information environment through which people repeatedly encounter particular goals, food choices, and action plans.

Public-health concerns arise both from the quality of individual dietary items and from the way content is repeatedly organized on a platform. Online dietary advice varies in accuracy, readability, and evidence presentation (6, 7), while diet, nutrition, and health content may contain weak evidence, exaggerated promises, or incomplete applicability conditions (8). Selective-exposure and health-information echo-chamber research further indicates that users do not meet all health information evenly; headlines, content cues, similarity, and information fatigue shape what is processed (9, 10). Existing work has therefore begun to move from single items toward information environments, but it has rarely examined whether visible platform content becomes concentrated around particular goals, food or method pathways, and conditions of applicability. This study uses an explicit distinction: topical concentration created by a keyword is not, by itself, structural narrowing. Narrowing is considered only when several features, including goal concentration, method compression, object substitution, templating, or weakened applicability boundaries, appear together within the same entry.

## 2 Literature review

### 2.1 Conceptualizing platform information structure

Existing online nutrition research has mainly evaluated individual items, providing the starting point for this study. Reviews of online nutrition content analysis commonly focus on accuracy, quality, and information sources (6), while readability and evidence presentation determine whether dietary advice can be understood and judged by the public (7). Studies of Chinese weight-loss videos, Instagram nutrition influencers, and TikTok food-diary content likewise examine accuracy, reliability, or engagement within particular content types (11–13). Other work has documented incomplete information, deceptive expression, and potential health harm (8). Research on psoriasis, pregnancy nutrition, gluten-free diets, diabetes self-management, inflammatory bowel disease, ulcerative-colitis surgery, NAFLD, and PCOS shows that target populations, risk warnings, and professional boundaries become especially important when dietary advice enters disease, bodily-indicator, or physiological-state contexts (14–22). These studies identify weaknesses within specific items, but they say less about whether public platform content is repeatedly organized into stable structural patterns.

Research on public healthy-eating content has also highlighted body-management goals, productized objects, and recurring expressive forms, but these strands are rarely integrated. Diet-culture and fitspiration studies show how weight goals enter dietary and body-management discourse (23, 24); supplement research foregrounds commercial objects and related risks (25, 26); and work on dietary guidance for cancer survivors on Xiaohongshu shows that care narratives, experiential authority, and commercial elements can coexist (27). Together, these findings raise a broader question. When similar goals, methods, products, and applicability boundaries recur within one platform, does the apparent abundance of content conceal a narrower set of usable dietary meanings? The present study addresses that question at the level of public information structure rather than single-item accuracy.

*RQ1*: What overall structure do public healthy eating posts display in terms of health goals, food or method pathways, and conditions of applicability?

### 2.2 Search terms and structural differentiation

Search entries are more than sampling devices. They are semantic routes through which users enter a platform’s healthy-eating information environment. Research on selective exposure shows that headlines, content, and presentation cues shape whether people choose to open health information (9). Studies of Chinese youths’ use of fitness-related information further point to the combined roles of intrinsic needs, perceived algorithms, and source credibility (28), while research on personalized health-information seeking suggests that users organize information needs around concrete health goals (29). Users therefore encounter local content collections through particular needs, terms, and platform cues rather than through a single undifferentiated pool.

Social-media diet research is often organized around one hashtag, topic, or disease context. Work on diet culture, fitspiration, “What I Eat in a Day” content, supplements, psoriasis diets, pregnancy nutrition, and gluten-free diets has revealed several features of platform diet communication (13–16, 22–26), but rarely compares their structural distribution across search entries. If entry semantics are associated with different goals, action pathways, or disclosure practices, they may expose users to different local information environments. This motivates the second research question.

*RQ2*: Does the information structure of public healthy eating content show differentiated narrowing across different search terms?

### 2.3 Public interaction and structural visibility

Public interaction adds a visibility layer to platform dietary information. Likes, comments, saves, shares, and replies are commonly used to describe public response to food and nutrition content (30), and engagement metrics are also used to examine the dissemination of health misinformation (31). These counts are not measures of actual exposure, algorithmic ranking, or adoption, but they can indicate which content structures receive stronger visible response.

Interaction is not a dependable proxy for content quality. Research on nutrition information on Twitter has found that quality and sharing practices may diverge (32), and TikTok studies likewise report that accuracy, evidence, and engagement do not always move together (13). Chinese short-video research also places treatment-misinformation risk and interaction performance in the same analytical field without treating them as equivalent (33). The relevant question here is therefore whether highly interactive posts make particular goals, methods, or applicability patterns more visible.

Interaction counts provide a second way to examine information structure. The unique-post corpus describes content supply, search-entry comparisons show how semantic routes differ, and public interaction indicates which structures attract stronger visible response. RQ3 therefore asks whether highly interactive posts are more concentrated around particular goals, food or method pathways, or condition-disclosure patterns.

*RQ3*: Is highly interactive content more concentrated around particular health goals, food or method pathways, or condition-disclosure patterns?

### 2.4 Overall research questions and study positioning

Taken together, prior work provides three foundations for studying platform healthy-eating information. Online nutrition-quality research focuses on accuracy, readability, evidence disclosure, and misinformation risk (6–8, 11–13). Social-media diet research identifies weight goals, body ideals, supplement products, templated food records, and bodily-indicator or disease-state advice (13–27). Health-information seeking and interaction research shows that users enter local information environments through specific needs and cues, and that public response differs across content (9, 28–36).

The unresolved issue is structural. Item-level studies do not show whether similar content is repeatedly organized into a small set of stable types. Topic-specific platform studies reveal important fragments but do not integrate them into a single description of the same information environment. Search and interaction studies explain entry and response, yet seldom compare the internal structure of different search routes. This study therefore describes public healthy-eating content as an information environment, compares its organization across search entries and interaction levels, and treats structural narrowing as a combination of features rather than as a synonym for topical focus.

## 3 Materials and methods

### 3.1 Study object and scope of interpretation

This study examines publicly searchable healthy-eating image-text posts that can be opened and read under stratified search entries. The unit of analysis is one public Xiaohongshu image-text post, including its title, body text, hashtags, OCR text from images, identifiable food objects, and visible interaction counts. The analysis focuses on lifestyle-management content related to daily eating, weight/body-shape management, bodily indicators or disease states, dietary methods, product substitution, and meal-plan templates. Author identities, user profiles, private messages, comment threads, personalized recommendation paths, and clinical accuracy assessment were outside the collected material.

This scope supports a content-structure diagnosis of visible search-entry environments. Public likes, saves, and comments are used as response indicators for comparing structures across entries, while questions about algorithmic causation, actual exposure, dietary behavior change, or individual health harm require different data designs. The discussion of an informationally partial environment is therefore limited to structural cues in publicly visible search-entry content.

### 3.2 Search-entry stratification and sample acquisition

Search-entry stratification was designed to compare semantic routes, not to reconstruct the platform-wide distribution of healthy-eating content. The six routes covered general healthy eating, weight/body-shape management, bodily indicators or disease states, method mechanisms, product substitution, and templated arrangements. Each route contained four original Chinese keywords, and 25 qualifying public image-text posts were sampled per keyword. Data were manually accessed through the Xiaohongshu mobile application using the standard search function from 28 July 2026 at 17:56:43 to 30 July 2026 at 13:25:47. Duplicate views encountered within the same keyword search were excluded during collection. When the same post appeared under different keywords, each occurrence was retained for entry-level comparison, but the post was represented once in the unique-post corpus. Video, livestream, clearly irrelevant, inaccessible, and otherwise non-qualifying material was excluded. This procedure produced 600 search-occurrence records and, after deduplication and manual review, 594 unique core posts. The occurrence corpus was used for entry comparisons, while the unique-post corpus was used for overall structure and high-interaction analyses. Because the aim was semantic stratification rather than ranking reconstruction, result-page numbers, positions, and fixed scrolling counts were not recorded and are not used to infer search ranking or recommendation strength (Tables 1, 2).

**Table 1:** Search-entry stratification and keyword settings.

Entry type	Keywords	Entry semantics	Rationale	Main exclusion rules
General healthy eating entry	健康饮食 (healthy eating); 营养搭配 (nutrition pairing); 合理膳食 (balanced diet); 科学饮食 (scientific eating)	Broad reference, with fewer prior assumptions about weight, products, or methods	To observe whether a general healthy eating entry retains balanced, diverse, and guideline-oriented expression	Posts limited to ordinary meal sharing, restaurant visits, lifestyle records, or content without a healthy eating goal were excluded
Weight/body-shape management entry	减脂餐 (fat-loss meals); 低卡饮食 (low-calorie diet); 瘦身餐 (slimming meals); 减脂食谱 (fat-loss recipes)	Strong goal presupposition, pointing to fat loss, weight reduction, and body shaping	To observe whether weight goals compress healthy eating expression and drive restriction-control pathways	Posts dominated by exercise, aesthetic medicine, clothing/body display, or non-diet content were excluded
Bodily indicator/disease-state management entry	控糖饮食 (glucose-control diet); 低GI饮食 (low-GI diet); 高血压饮食 (hypertension diet); 降尿酸饮食 (uric-acid-lowering diet)	Strong state presupposition, placing diet in indicator or chronic-disease management contexts	To observe whether bodily indicator/disease-state dietary content creates pressure for condition disclosure	Posts on medication, medical consultation, or disease education without dietary management content were excluded
Method-mechanism entry	轻断食 (intermittent fasting); 16 + 8 (16:8); 碳循环 (carbohydrate cycling); 低碳水 (low carbohydrate)	Strong method presupposition, foregrounding action rules	To observe whether healthy eating is reorganized as rule-based or cyclical schemes	Posts containing only a challenge name, slogan, or method label without observable dietary mechanism content were excluded
Product-substitution entry	代餐 (meal replacement); 蛋白粉 (protein powder); 营养补剂 (nutritional supplements); 代餐食品 (meal-replacement food)	Strong object presupposition, highlighting products, supplements, or functional foods	To observe whether healthy eating is substituted by productized objects and whether this creates pressure for risk disclosure	Posts that were only reviews, unboxing, or product seeding without dietary mechanism content were excluded
Templated entry	一周餐单 (weekly meal plan); 照着吃 (eat as shown); 一日三餐安排 (three-meal arrangement); 饮食打卡 (diet check-in)	Strong form presupposition, highlighting replicable arrangements	To observe whether healthy eating is formatted through meal plans, plans, and check-in routines	Posts without a concrete meal plan, without replicable arrangements, or only using related terms loosely were excluded

**Table 2:** Sample acquisition and screening workflow.

Stage	Purpose	Main operations	Recorded fields	Not handled at this stage
Entry search and candidate collection	Build a candidate pool for predefined entries	Enter the original Chinese keyword in the platform search function; exclude video, livestream, clearly irrelevant, inaccessible, and within-keyword duplicate material; retain qualifying public image-text posts until 25 posts are obtained for each keyword	candidate_id; search_keyword; capture_time; title; body; tags; publish_time; note_type_precheck; raw_link_check; pre_like_text; pre_collect_text; pre_comment_text	No result-page number, ranking position, fixed scroll count, or page-skipping log was recorded; candidate interaction text was not used as a formal interaction variable
Rapid screening	Determine whether a candidate enters formal coding	Use the title, body, tags, and visible images to assess relevance to healthy eating, dietary management, glucose control, weight management, nutrition pairing, recipes, meal plans, or food choice; remove non-core or indeterminate material	quick_health_diet_relevance; quick_exclusion_flag; quick_exclusion_reason; candidate_status	No G/F/M/T/Q/A decision, complete OCR, or judgment of advice boundaries was made at this stage
Formal coding and quality review	Verify materials and code the retained sample	Check the source material, image-text status, usable image, OCR text, and visible interaction bar; complete G/F/M/T/Q/A coding and record quality-review information	sample_status; material_quality; review_flag; review_type; core_usable; G_primary; F1-F8; M_primary; T_template; Q_code; A matrix; A_code	No author, avatar, profile, user ID, note ID, or other traceable field was collected; isolated interaction numbers were not entered as formal variables
Analysis-sample generation	Separate entry comparisons from the unique-post corpus	Retain 600 search-occurrence records for entry comparisons; retain cross-keyword or cross-entry repeats as occurrence records, then deduplicate them to form 594 unique core posts for overall and interaction analyses	search-occurrence record; unique post; entry_group; search_keyword; repeated-retrieval flag; core-analysis flag	Repeated retrievals were not interpreted as evidence of recommendation, algorithmic exposure, or search ranking

### 3.3 Coding dimensions and rationale

The coding framework followed content-analysis principles (37) and covered three structural dimensions: health goals, food or method pathways, and applicability conditions. These dimensions were operationalized through six coding fields. The goal field captured the outcome toward which healthy eating was directed. The food or method fields recorded basic food groups, productized objects, dominant methods, and templated presentation. The applicability fields captured advice or claim triggers and the extent to which relevant conditions were disclosed. Together, they describe how lifestyle-management information is organized in a public digital-health setting. Existing social-media studies have examined videos, Instagram posts, TikTok content, disease- or bodily-state-related dietary information, supplement communication, and platform diet narratives through themes such as quality, accuracy, reliability, interaction, risk warnings, and commercialization (11–26). Health-information assessment tools also emphasize source information, understandability, actionability, and boundaries around advice (38–40). Basic food-group categories were based on the Chinese Dietary Guidelines (2022) and related balanced-diet and plate illustrations (41–43). Criteria for risk warnings, target populations, exaggerated claims, commercial identification, and product warnings were based on the relevant Chinese health-popularization and advertising provisions (44–47).

Rather than replacing DISCERN, JAMA benchmarks, or PEMAT, which assess individual health-information items, these fields shift the analysis toward the distribution of visible content. Considering goals, methods, objects, templates, and applicability conditions together makes it possible to distinguish ordinary entry focus from a broader combination of narrowing features (Table 3).

**Table 3:** Coding dimensions, rules, and study functions.

Dimension	Variable code	Coding type	Core decision rule	Rationale	Function in this study
Health goal	G	Single-choice	The primary goal was judged from the title, main claim in the body text, hashtags, and effect claims; categories included general health/balance, weight/body shape, bodily indicator/disease state, and no explicit core goal	Social media diet and health information studies suggest that health goals organize content meaning (14–24, 28, 29, 34–36)	To measure goal concentration
Food-category coverage	F1-F8	Multiple-choice	F1-F7 record basic food groups; F8 separately records products, supplements, meal replacements, or functional foods	Dietary guidelines, the balanced diet pagoda, and the plate illustration provide the boundaries of basic food groups (41–43)	To measure food coverage and productized objects
Main method pathway	M	Single-choice	Categories included balanced pairing, restriction/control, time/rhythm, product substitution, and no explicit method; coding followed the dominant action mechanism	Social media diet content often organizes eating through rules, rhythms, or substitution plans (11–26)	To measure method-pathway concentration
Templated presentation	T	Binary	Posts with meal plans, schedules, “eat as shown,” check-ins, “copy this,” or other replicable arrangements were coded as 1	Templating is an important form of platform healthy eating content (13, 23, 24)	To measure action formatting
Advice/claim trigger	Q	Binary	Content that addressed readers with advice, instructions, recommendations, or effect claims was coded as Q1; pure record or display content was coded as Q0	Health popularization regulations, internet advertising rules, and health-food warning rules all address boundaries around advice, recommendations, and claims (44–47)	To identify content requiring further condition assessment
Condition-disclosure status	A	Assessed only within Q1 posts	Completeness, partiality, or absence of conditions was judged according to population, scenario, dosage, frequency, duration, risks/contraindications, absolute claims, and object generalization; the resulting categories were A1 (relatively complete), A2 (partial), and A3 (missing).	Health popularization regulations, internet advertising rules, and health-food warning rules jointly emphasize target population, risk warnings, and avoidance of exaggerated claims (44–47)	To measure sufficiency of advice boundaries

### 3.4 Indicator construction and analysis strategy

The main analysis used unweighted content structures. For the single-choice variables G and M, category proportions, Top1, Top3, HHI, and normalized entropy were reported. HHI = sum(s_i^2), where s_i is the share of category i. Values closer to 1 indicate concentration in a small number of categories. Normalized entropy = −sum(s_i ln s_i)/ln(k), where k is the number of effective categories. Values closer to 1 indicate a more even distribution and values closer to 0 indicate stronger concentration (48–50). Because F is a multiple-choice variable, it was summarized through F1-F7 basic-food coverage and the F8 productized-object rate rather than the same entropy index. T, Q, and A were reported as proportions, with A calculated only among Q1 posts so that ordinary display content was not treated as condition-deficient. These measures describe the constructed stratified corpus and were not weighted to represent platform-wide distributions, search positions, or exposure probabilities.

Three supplementary analyses were conducted. Structural combinations joined G, M, T, and F8 to examine whether an entry was occupied by a small number of goal-method-template-product configurations. The Top3 share used valid G x M x T x F8 records within each entry as its denominator. Cramer’s V was paired with chi-square tests to describe exploratory associations between entry type and structural variables, with sample size, degrees of freedom, and *p* values reported in the results. Public interaction was defined as likes plus saves plus comments, and keyword means were calculated from this total. The high-interaction group comprised the upper quartile of total interaction counts among the 594 unique core posts. Zero values were retained as observed zeros, while missing or unverified interaction values were excluded from interaction analyses. These comparisons describe visible response patterns and do not establish exposure, recommendation, or adoption (Table 4).

**Table 4:** Main indicators, analysis units, and interpretive scope.

Indicator	Analysis unit/denominator	Main use	Interpretive scope
HHI, normalized entropy	G and M single-choice variables in the 594 unique core posts	Describe concentration in goals or methods	Describes the constructed corpus; does not infer platform algorithms or user behavior
F1-F7 coverage, F8 rate	594 unique core posts; entry comparisons use the corresponding entry sample	Describe basic-food breadth and productized-object occurrence	Does not assess the medical correctness of an individual recommendation
Top3 structural-combination share	Valid G x M x T x F8 records within each entry	Describe whether an entry is occupied by a few recurring combinations	Describes entry-level concentration; does not represent all platform content
Cramers V and chi-square	Cross-tabulations of entry type and structural variables, *N* = 600 search-occurrence records	Describe exploratory association strength and its test statistics	Does not establish the source of association, causality, or algorithmic mechanism
High-interaction group difference	594 unique core posts; upper quartile *N* = 149 and remainder *N* = 445	Describe structural differences in posts with stronger visible response	Does not substitute for evidence of exposure, ranking, recommendation, or adoption

The 600 records are search-occurrence records used for entry comparisons. Overall structure, high-interaction comparisons, and deduplicated entry summaries use 594 unique core posts. The entry-level core usable counts were 100, 100, 100, 99, 95, and 100 for general healthy eating, weight/body-shape management, bodily indicator/disease-state management, method-mechanism, product-substitution, and templated entries, respectively. No weighting was applied to the pooled descriptive analyses.

### 3.5 Quality control

During collection, candidate material was screened for video, livestream, comment-section images, ordinary meal sharing, restaurant visits, non-diet-dominant content, and material that could not be judged. Formal coding then covered G, F, M, T, Q, A, OCR text, image-based food identification, and interaction-bar verification. When a post triggered more than one goal, method, or boundary state, the decision followed a fixed priority order based on the title, the main claim in the body, repeated promises, calls to action, and image evidence. Images were used to identify food objects and visible text, but not to infer a health goal or advice claim on their own. Coding consistency was assessed through two paired coding rounds using Cohen’s kappa (68). The first round followed the frozen codebook, and the second was completed manually without referring to the first round’s item-level labels; the two records were aligned by final_sample_id. Across 600 paired search-occurrence records, Cohen’s kappa for the core fields ranged from 0.794 to 1.000. Among the 590 records classified as core usable by both rounds, the range was 0.863 to 1.000. The 590-record common-core subset was used only for the reliability comparison and is not a separate substantive analysis sample. For G_primary, M_primary, T_template, Q_code, and A_code, the full-sample kappas were 0.974, 0.971, 1.000, 0.973, and 0.961, respectively. After formal coding, the study checked valid values, abnormal combinations, the logical relation between Q and A, and consistency in the interaction fields (Table 5).

**Table 5:** Inter-coder agreement results.

Coding dimension	Variable or field	Full-sample Kappa	Common-core Kappa	Sample size	Note
Health goal	G_primary	0.974	0.980	600; 590	High agreement in primary-goal classification
Food categories	F1-F8	0.884–0.993	0.912–0.996	600; 590	F4 showed the lowest field-level agreement
Main method pathway	M_primary	0.971	0.981	600; 590	High agreement in dominant-method classification
Templated presentation	T_template	1.000	1.000	600; 590	Complete agreement across both samples
Advice or claim trigger	Q_code	0.973	0.980	600; 590	High agreement in identifying reader-directed advice or claims
Applicability status	A_code	0.961	0.972	600; 590	Agreement in the composite condition-disclosure judgment
Applicability details	A_person and seven related fields	0.794–1.000	0.863–1.000	600; 590	Dosage and duration showed relatively more disagreement

Kappa denotes Cohen’s kappa. The full sample includes 600 paired search-occurrence records. The common-core sample includes 590 records coded as core usable by both rounds. Complete field-level results and confusion matrices can be provided as Supplementary material.

## 4 Results

The results proceed from the pooled structure of the entry-stratified corpus to differences between search entries, structural combinations, and public interaction. Overall structure and interaction analyses use the 594 unique core posts. Entry comparisons use the 600 search-occurrence records.

### 4.1 Overall structure: goals and methods concentrate before food objects

The six entry types yielded 594 unique core posts after deduplication. General healthy eating, weight/body-shape management, bodily indicator/disease-state management, and templated entries each contributed 100 posts, while the method-mechanism and product-substitution entries contributed 99 and 95, respectively. These figures describe the constructed stratified corpus rather than the platform-wide distribution, and the modest imbalance is reported explicitly in the relevant table notes.

In the pooled unique-post corpus, concentration appeared first in health goals and method pathways. Weight/body-shape orientation accounted for 66.33%, bodily indicator/disease-state orientation for 22.90%, general health/balance for 6.40%, and display/record content for 4.38%. The goal Top1 and Top3 shares were 66.33 and 95.62%, with an HHI of 0.4984 and normalized entropy of 0.6656. For method pathways, restriction/control accounted for 40.07%, no explicit method for 19.36%, balanced pairing for 22.39%, time/rhythm for 11.78%, and product substitution for 6.40%. The method Top1 and Top3 shares were 40.07 and 81.82%, with an HHI of 0.2661 and normalized entropy of 0.8993. The sample was therefore more concentrated in the purpose of eating than in the specific way of eating (Figure 1).

**Figure 1:**
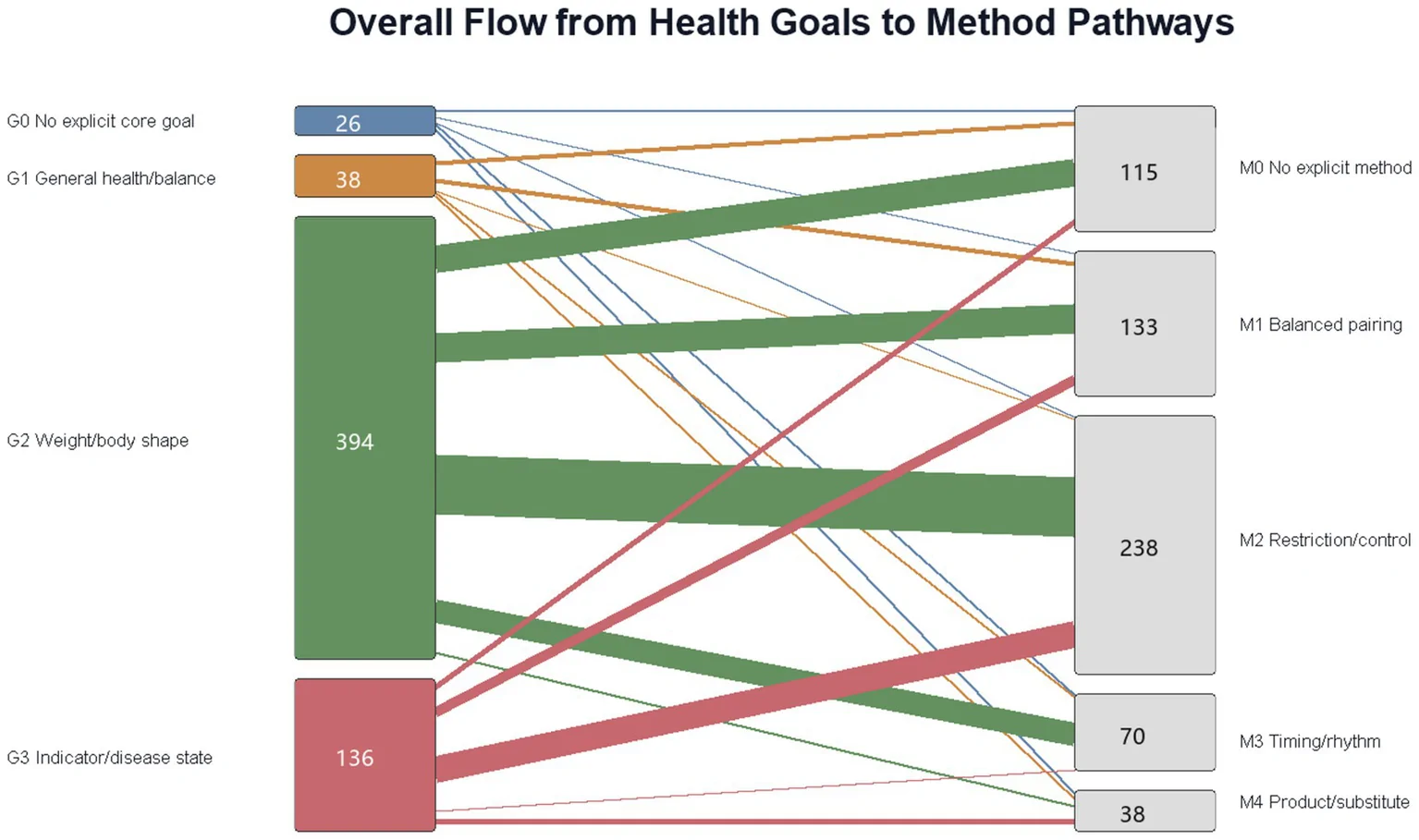
Flow from health goals to method pathways in the overall sample.

Food objects retained substantial diversity while being repeatedly organized through weight, control, rhythm, template, and product-substitution schemes. The mean coverage of the seven basic-food groups was 3.75 categories, the basic-food coverage rate was 53.61%, and F8 product, supplement, or meal-replacement objects appeared in 20.14% of posts. Templated presentation appeared in 52.86% of posts and Q1 advice or claim triggers in 47.14%. Within Q1 posts, A1 relatively complete conditions accounted for 63.21%, whereas partial conditions (A2) plus missing conditions (A3) accounted for 35.71%. These values capture the completeness of applicability disclosure and map structural positions in the corpus. Taken together, the findings describe a reorganization of healthy eating around goals, methods, formats, and applicability boundaries alongside continued diversity in food objects.

### 4.2 Entry differences: different entries show different types of narrowing

To answer RQ2, the analysis compared goals, methods, food categories, and applicability conditions across entries. The patterns differed by semantic route. In the weight/body-shape entry, G2 accounted for 94.00%, M2 restriction/control for 61.00%, and F1-F7 basic-food coverage for 39.43%. The entry therefore showed more than the expected topical focus of fat-loss terms. It combined a concentrated weight goal with a restriction/control pathway and lower basic-food coverage. In the bodily indicator/disease-state entry, G3 accounted for 90.00%, M2 for 66.00%, Q1 for 59.00%, and partial conditions (A2) plus missing conditions (A3) among Q1 posts for 44.07%. This combination points to a stronger need for explicit target-population, monitoring, and risk information. These A2/A3 patterns indicate incomplete disclosure of applicability conditions, not verified medical errors, legal violations, or health harm (Figures 2, 3).

**Figure 2:**
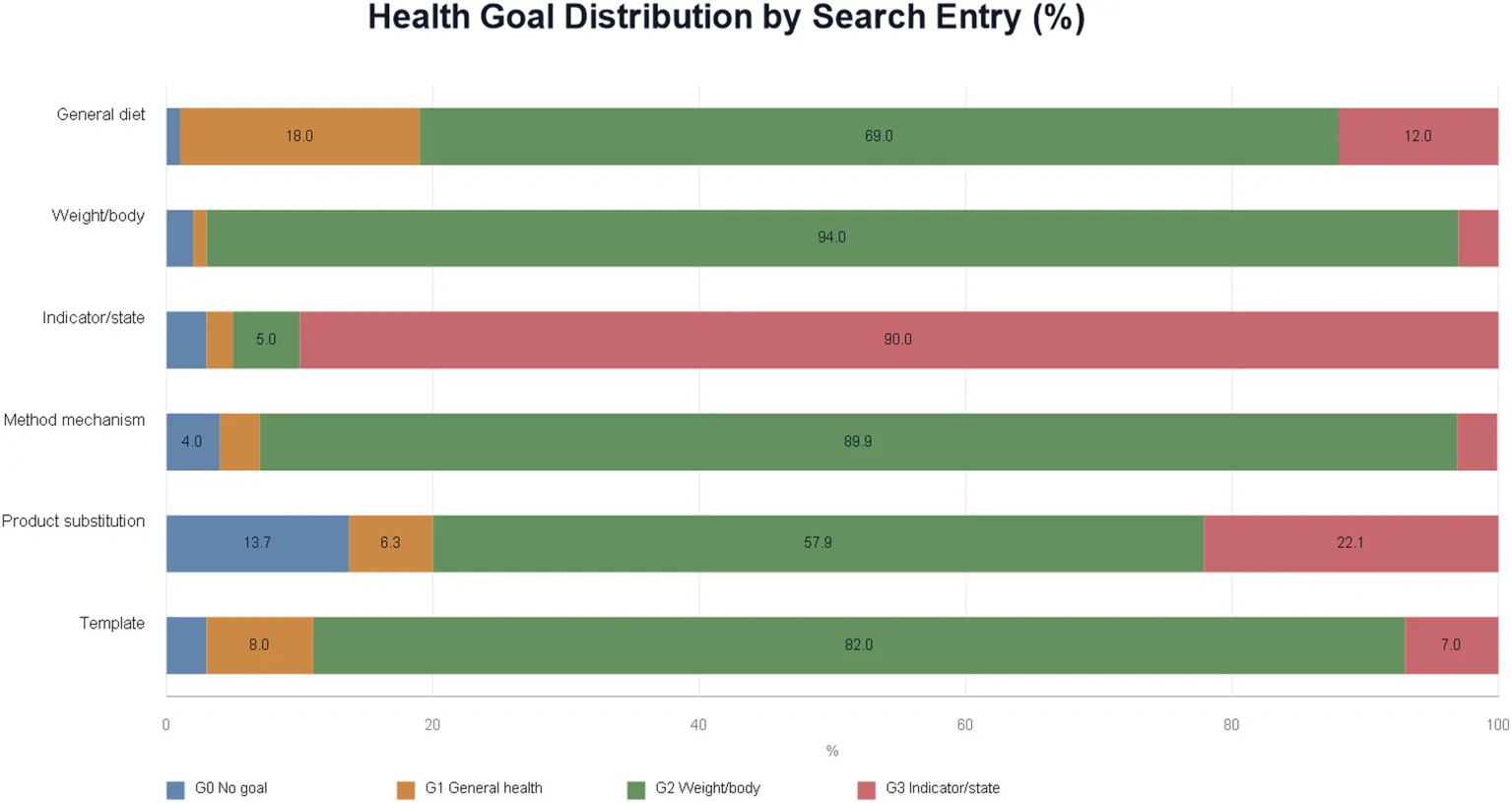
Distribution of health goals across entries.

**Figure 3:**
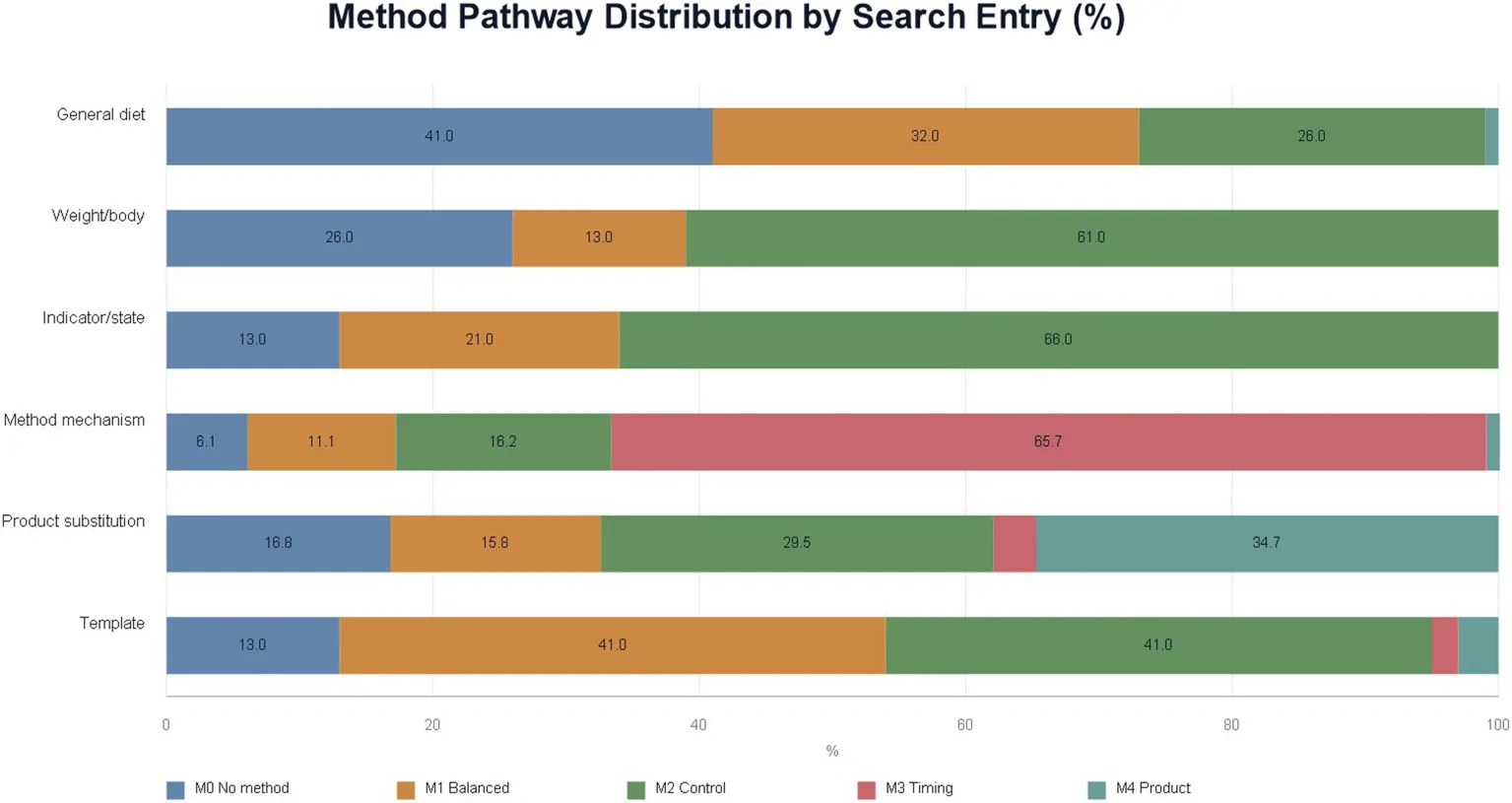
Distribution of method pathways across entries.

The method-mechanism entry showed another configuration. M3 time/rhythm or scheme mechanisms accounted for 65.66%, templated presentation for 76.77%, and F1-F7 coverage for 73.34%. Intermittent fasting, 16:8, carbohydrate cycling, and low-carbohydrate content therefore organized food choices through cycles, rhythms, or rules without necessarily reducing the range of food groups. The product-substitution entry showed object transfer. F8 objects appeared in 76.84% of posts, M4 product substitution accounted for 34.74%, F1-F7 coverage was 37.99%, Q1 was 64.21%, and partial or missing conditions (A2 + A3) among Q1 posts was 50.82%. Its narrowing was concentrated less in a single goal than in the joint presence of product objects, advice or claims, and weaker applicability boundaries. In the templated entry, T was 72.00%, G2 was 82.00%, and M1 balanced pairing and M2 restriction/control each accounted for 41.00%. The entry converted healthy eating into followable arrangements without necessarily making products central (Figures 4–6).

**Figure 4:**
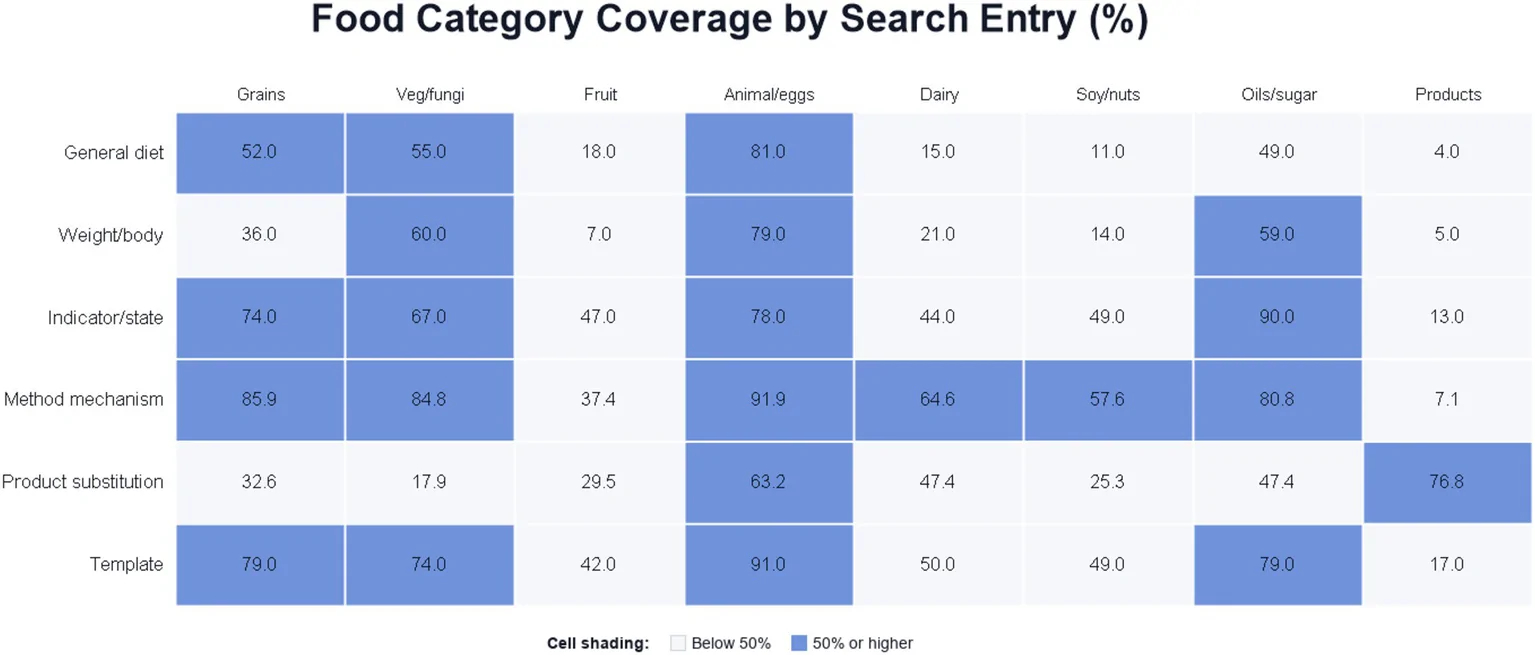
Food-category coverage across entries.

**Figure 5:**
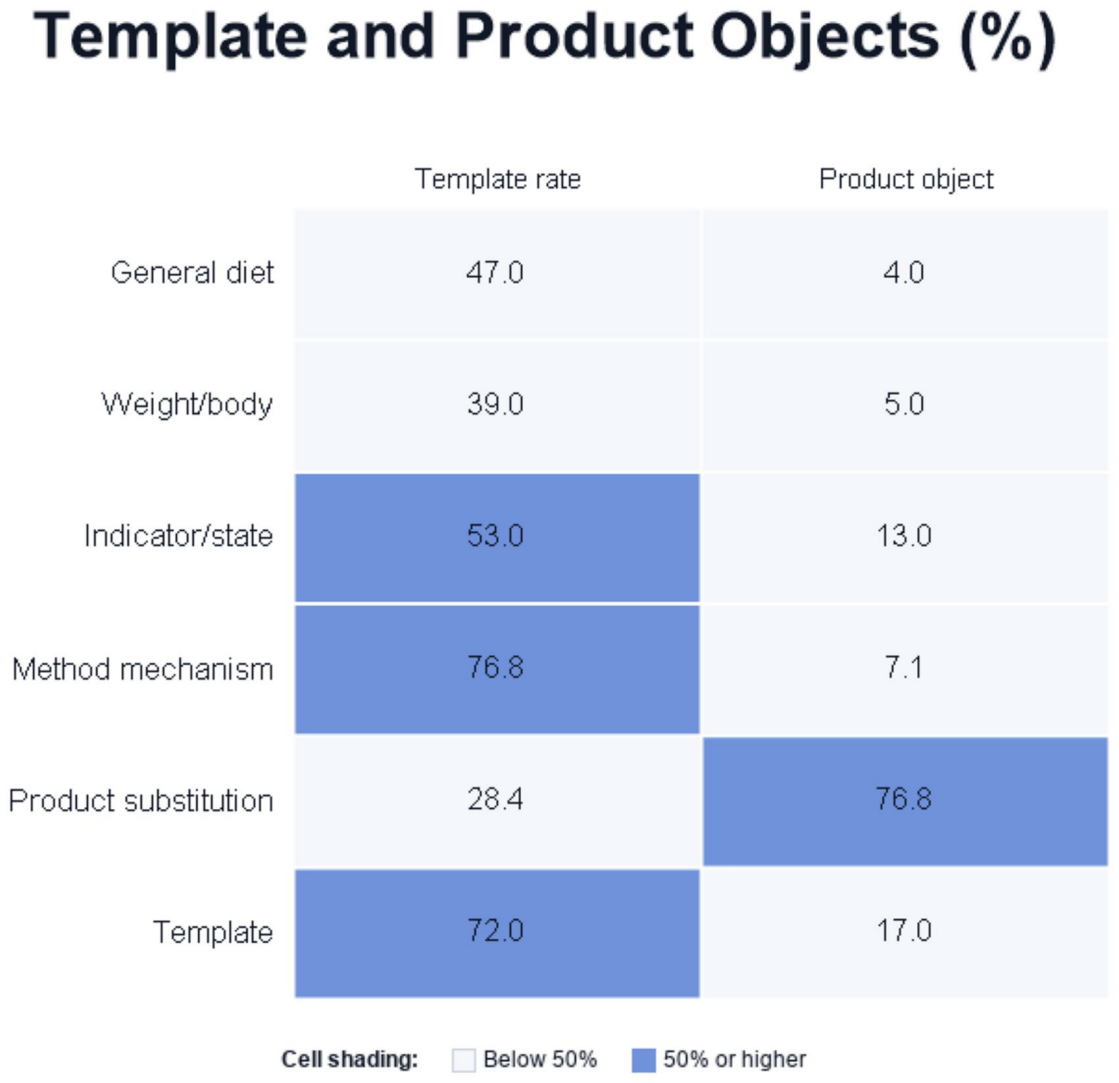
Templated expression and productized objects.

**Figure 6:**
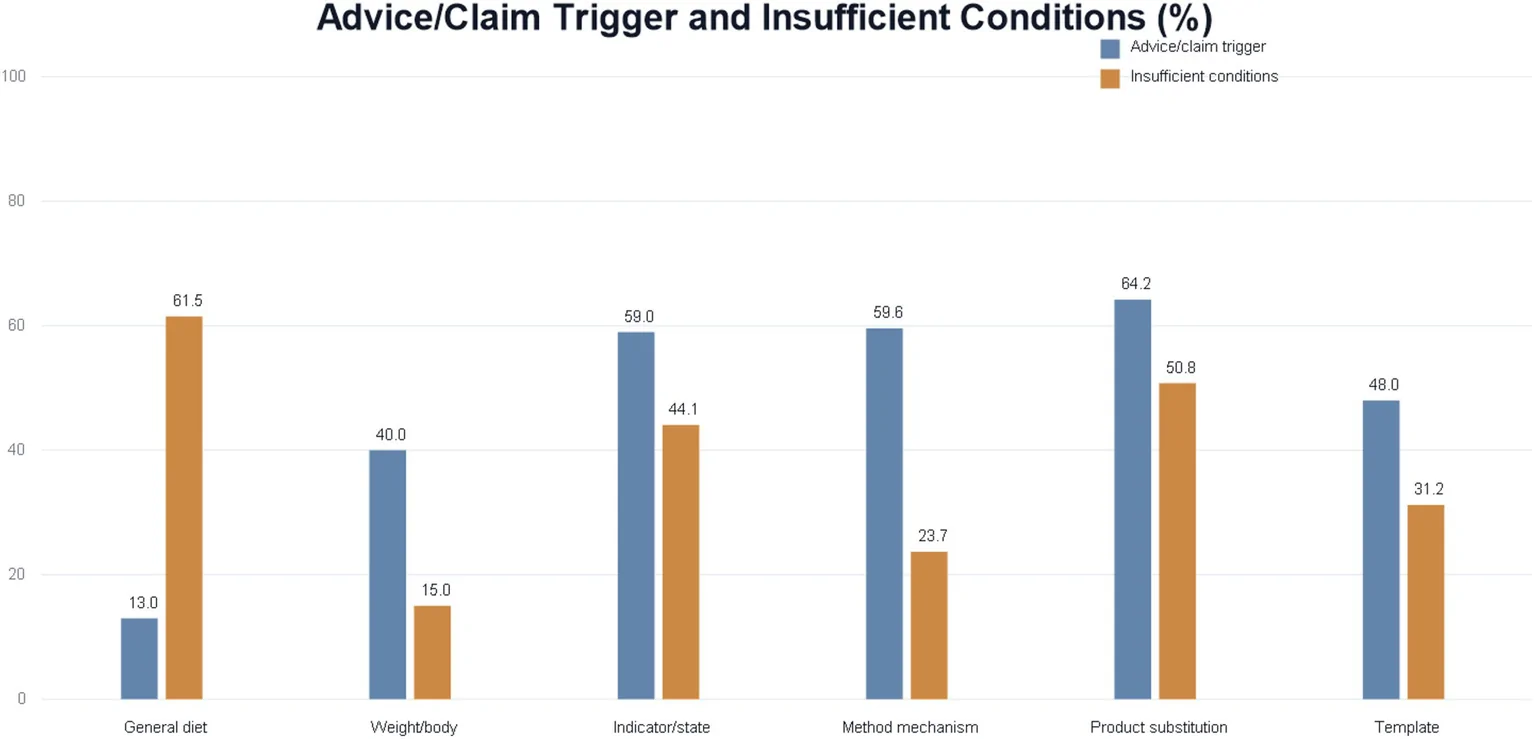
Advice/claim triggers and condition insufficiency.

Condition-detail analysis showed that risks and contraindications were the least frequently disclosed boundary element. Among Q1 posts, risk or contraindication coverage was 27.87% in the product-substitution entry, 18.64% in the bodily indicator/disease-state entry, 6.78% in the method-mechanism entry, 6.25% in the templated entry, 2.50% in the weight entry, and 0 in the general entry. Dosage coverage was generally higher, but frequency and duration remained weak in several entries. In the product entry, duration coverage was 68.85% and frequency coverage was 21.31%. The issue is therefore not a generic claim of low information quality. It can be located in specific omissions involving risks, contraindications, frequency, duration, target populations, and usage scenarios (Figure 7).

**Figure 7:**
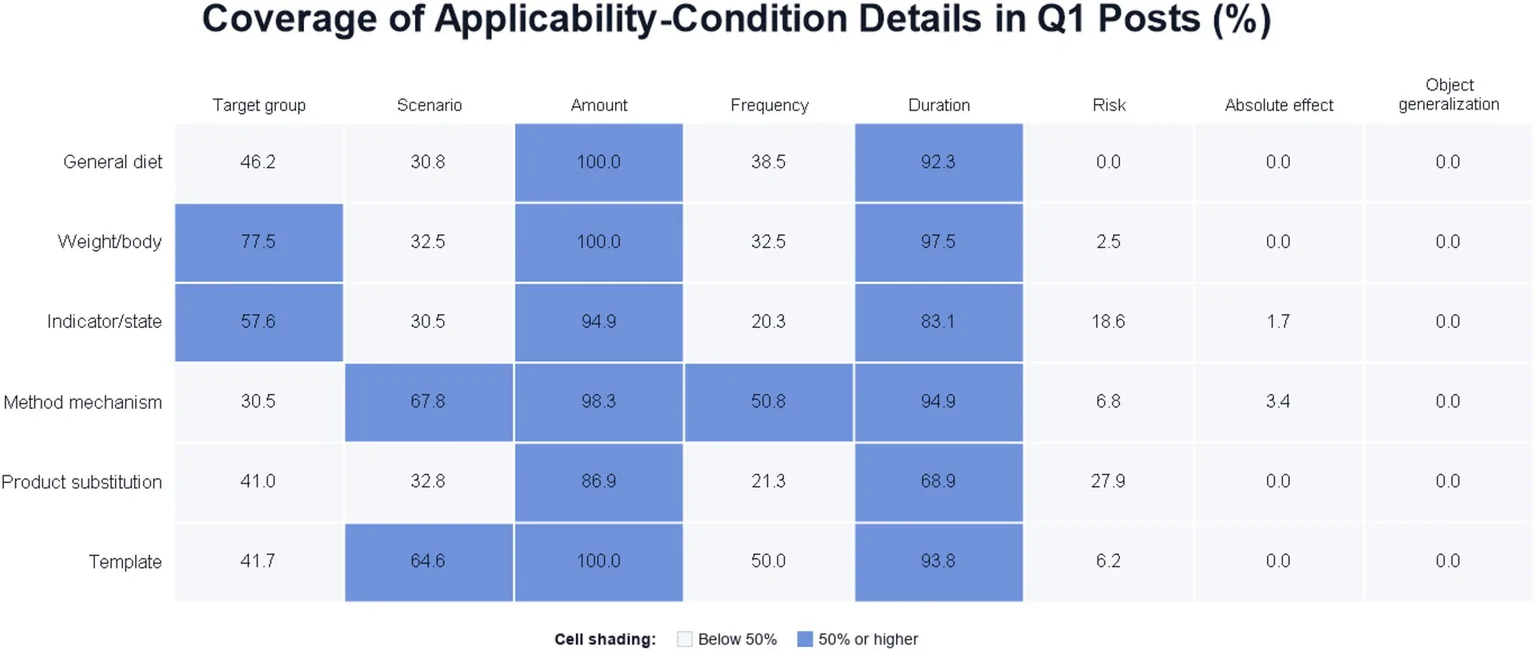
Coverage of condition details among Q1 posts.

Taken together, the weight/body-shape, bodily indicator/disease-state, method-mechanism, product-substitution, and templated entries represented different combinations of concentration, object substitution, formatting, and condition pressure. The results answer RQ2 by showing that entry semantics are associated with distinct structural configurations rather than one uniform form of narrowing.

### 4.3 Structural combinations: single dimensions underestimate entry-level narrowing

Single-dimensional comparisons reveal where entries differ, but they do not show whether the same features recur together. The combination analysis therefore joined health goals, method pathways, templating, and product objects. In the pooled sample, the three most frequent configurations were G2 weight/body shape with M2 restriction/control, templating, and no product object (12.79%); the same goal and method without templating or a product object (9.09%); and G2 with M3 time/rhythm, templating, and no product object (7.24%). All three centered on weight/body-shape goals, but they organized action through different methods and formats.

The Top3 share further separated the entries. It was 68.00% in the weight/body-shape entry, 66.67% in the method-mechanism entry, 63.00% in the bodily indicator/disease-state entry, 52.00% in the templated entry, 37.00% in the general healthy-eating entry, and 31.58% in the product-substitution entry. Weight, method, and indicator entries were therefore more internally concentrated. The product entry had the strongest product-object signal but more dispersed goal-method combinations, suggesting that its main pressure occurred at the object and applicability-boundary levels rather than through a few dominant combinations (Figure 8).

**Figure 8:**
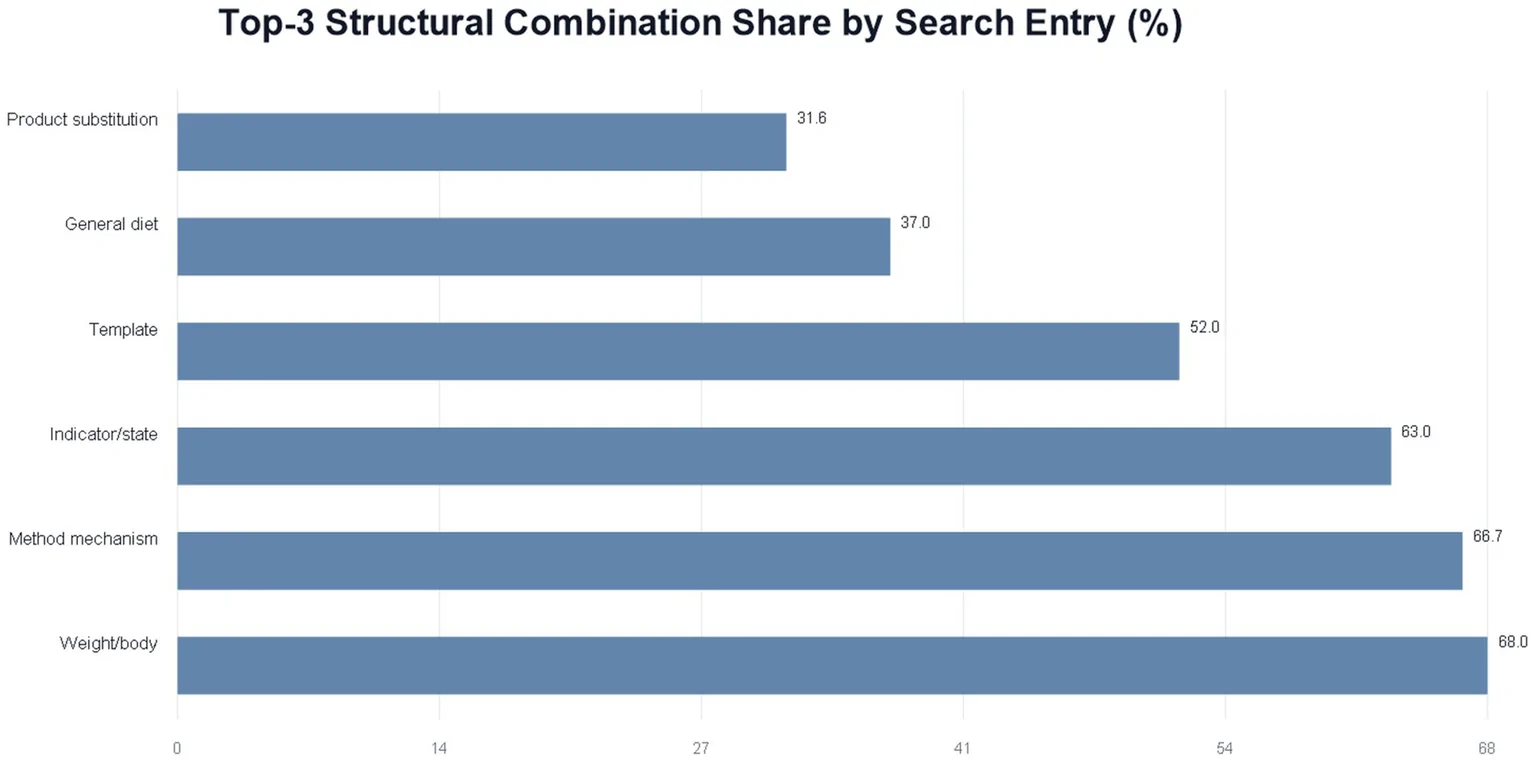
Top3 structural-combination share by entry. The Top3 share uses valid G x M x T x F8 combination records within each entry as the denominator.

The association results were consistent with this differentiation. Entry type had the strongest association with productized objects (Cramer’s V = 0.630), followed by method pathway (V = 0.484) and health goal (V = 0.464). Associations with advice or claim triggers (V = 0.348) and templating (V = 0.341) were weaker, while the reported association with condition-disclosure status was V = 0.213. The corresponding chi-square tests for G, M, T, Q, and A were significant in the 600-record occurrence corpus: G, chi-square (15) = 383.01; M, chi-square (20) = 556.75; T, chi-square (5) = 69.27; Q, chi-square (5) = 71.77; and A, chi-square (20) = 107.66, with *p* < 0.001 for all tests. Because A is assessed within posts that make advice or effect claims, this association is interpreted descriptively and does not indicate verified medical error, unlawful content, or actual health harm. These exploratory associations describe the constructed corpus and leave algorithmic mechanisms and user-level effects for designs with ranking, exposure, or user-trace data (Figure 9).

**Figure 9:**
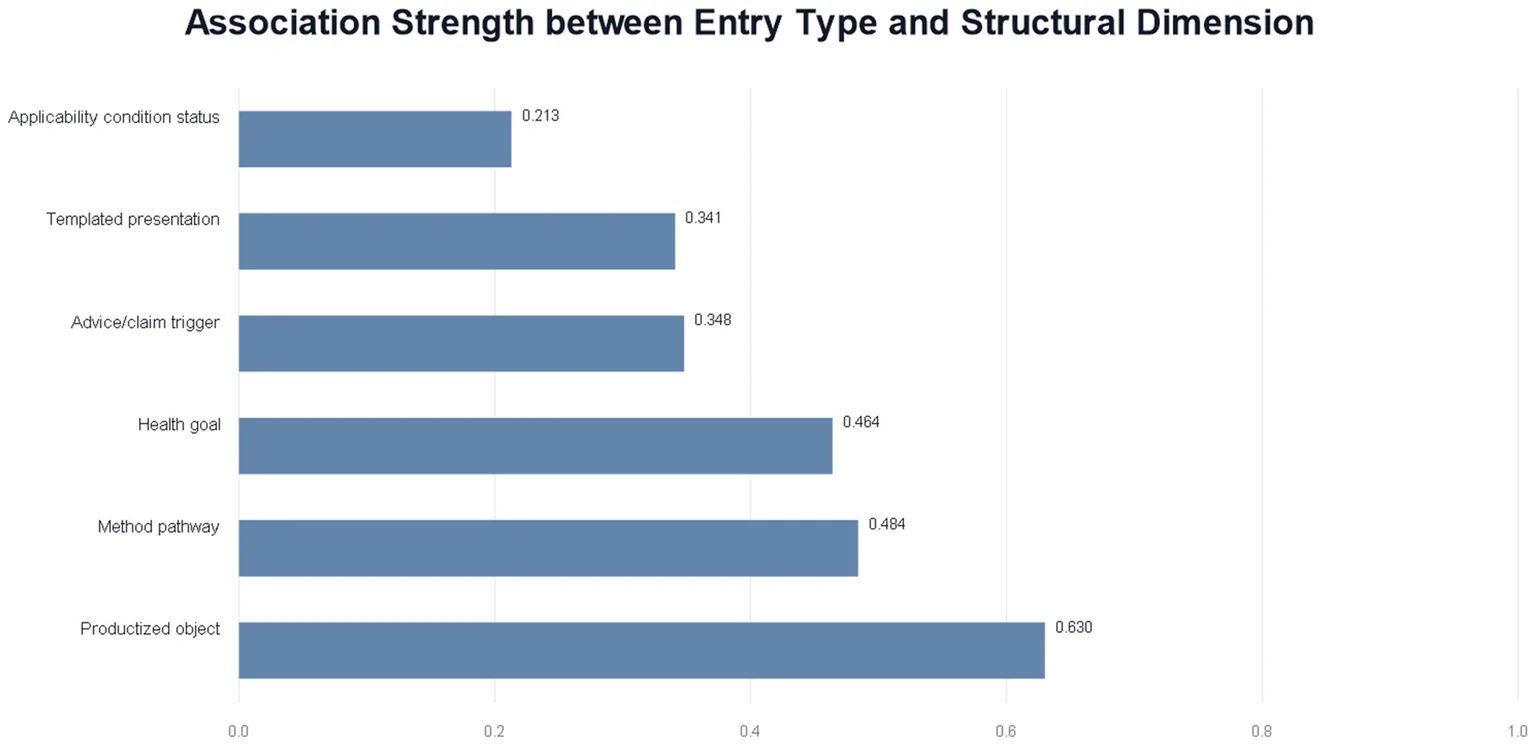
Association strength between entry type and structural dimensions. Cramer’s V and chi-square tests are based on 600 search-occurrence records and are interpreted as exploratory associations.

The combination analysis therefore adds information that single dimensions cannot provide. It shows not only that entries differ in their marginal distributions, but also that they organize goals, methods, templates, and product objects into different recurring configurations.

### 4.4 Public interaction counts: high interaction strengthens weight, control, and template structures

Public interaction also varied by entry and keyword. The mean across likes, saves, and comments was highest for the weight/body-shape entry (18,216.63), followed by the templated entry (8,554.55), the general healthy-eating entry (7,693.43), and the bodily indicator/disease-state entry (2,944.66). At the keyword level, fat-loss meals, weekly meal plans, fat-loss recipes, meal replacements, and slimming meals received the strongest visible response, whereas protein powder, diet check-ins, uric-acid-lowering diets, hypertension diets, meal-replacement foods, and nutritional supplements received less. Visible response was therefore concentrated around weight, fat loss, and replicable meal-plan routes rather than across all product or disease-state content.

The high-interaction comparison used the upper quartile of the total interaction count among the 594 unique core posts. The resulting group contained 149 posts, compared with 445 in the remainder. Weight/body-shape orientation accounted for 79.87% of the high-interaction group versus 61.80% of the remainder; restriction/control accounted for 44.97% versus 38.43%; and templated presentation accounted for 61.07% versus 50.11%. Productized objects were less common in the high-interaction group (9.40% versus 23.60%), as was partial or missing condition disclosure (14.09% versus 17.75%). Public response therefore clustered around weight, control, and templated structures without amplifying all boundary-related concerns (Figure 10).

**Figure 10:**
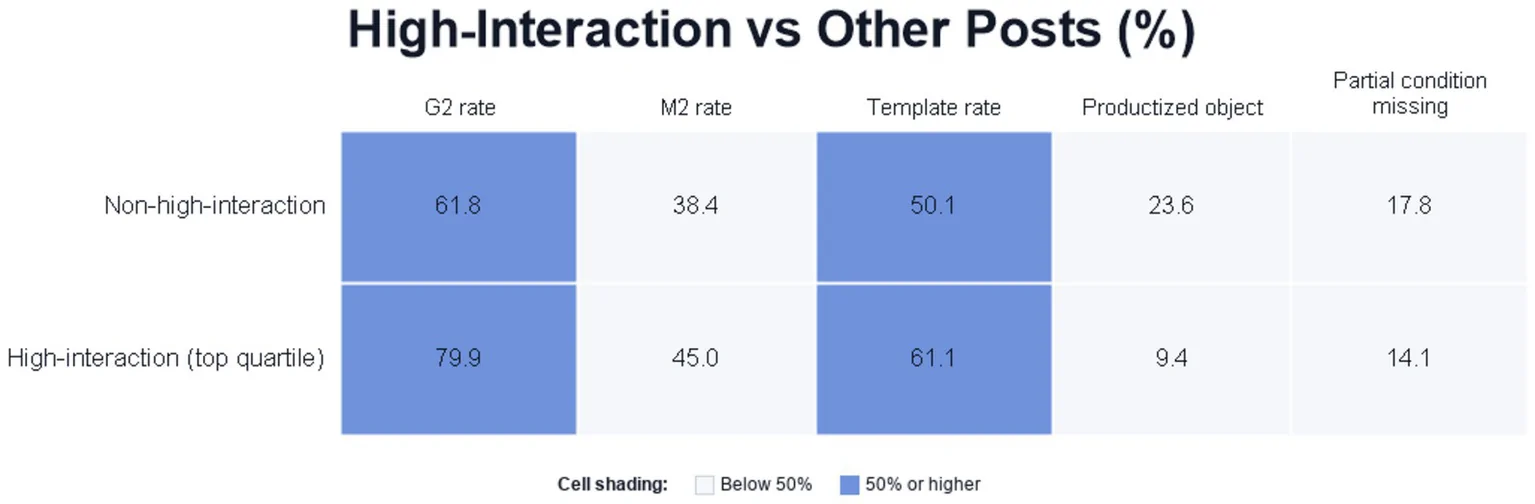
Structural differences between high-interaction and non-high-interaction groups. The high-interaction group is defined by the upper quartile of likes plus saves plus comments among the 594 unique core posts; *N* = 149 in the high-interaction group and *N* = 445 in the remainder.

RQ3 is answered by a qualified pattern. Highly interactive posts were more concentrated around weight/body-shape goals, restriction/control pathways, and templated presentation, but they did not show higher rates of productized objects or insufficient condition disclosure. The upper-quartile rule is an operational monitoring rule, not a validated measure of exposure or influence.

Three points summarize the results. First, concentration in the stratified corpus was strongest for goals and methods, not for food objects as such. Second, different entries expressed different combinations of goal concentration, method rules, object substitution, templating, and condition pressure. Third, public response was more visible around weight, control, and templates, but the data do not support treating high interaction as quality, recommendation, or adoption.

## 5 Discussion

### 5.1 Entry-based information environments and shared responsibility

The results place healthy eating information in localized entry-based environments. The weight entry is highly concentrated in goals and restriction pathways; the indicator entry combines state management with boundary pressure; the method entry turns healthy eating into rules and templates; the product entry highlights commercialized objects; and the template entry foregrounds formatted action. These entry differences show that healthy eating information on the platform has distinct local configurations and heterogeneous structural emphases.

This finding makes the allocation of responsibility for informational partiality more specific. Users’ keywords do contain intention: those who search for fat-loss meals expect weight-management content, and those who search for meal replacements may be more receptive to product objects. At the same time, the public content returned under an entry also reflects existing platform supply, creator templates, commercial promotional materials, and the way a search entry makes certain content more openable. Because the data do not include algorithm logs or personalized recommendation traces, the analysis locates this pattern in the publicly visible entry environment. The strong associations between entry type and productized objects, method pathways, and health goals show that users who keep searching through certain entries encounter clearly different versions of healthy eating.

This entry-based environment can be placed in dialogue with information fatigue while retaining a distinct analytical focus. Repeated exposure to similar information may lead to avoidance and fatigue (10), whereas this study observes the structural repetition of visible content under search entries. The former concerns how users are surrounded by information in flows; the latter concerns what public content combinations are offered when users actively search. Seen together, informational partiality can appear in personalized recommendation feeds and in active, goal-directed search scenarios. Platform governance can therefore examine recommendation algorithms together with repeated structural gaps in search entries, hashtags, and product-related content.

### 5.2 From single-item quality to entry-level governance

Fact-checking and item-level quality assessment remain foundations of digital nutrition governance. The quality and accuracy of online nutrition content are unstable (6), web-based dietary advice differs in readability and evidence presentation (7), and diet-health content may mislead through incompleteness, deceptive expression, or limited applicability boundaries (8). These concerns continue to call for professional review and corrective health communication. The present findings add an entry-level view: posts may appear as recipes, meal records, or experiential advice at the item level, while repeated presentation within an entry can direct healthy eating toward weight, control, fasting, meal replacement, and copyable templates. Repeated alignment between a goal and a method can therefore compress the public meaning of healthy eating across an entry.

Structural quality analysis identifies public health communication risks that can arise when content is accurate yet incomplete. High G2 and high M2 in the weight entry are expected because fat-loss meals, low-calorie diets, and fat-loss recipes are weight-goal entries. The diagnostic signal emerges when weight goals co-occur with restriction-control pathways and lower basic-food coverage. For the indicator entry, attention centers on whether bodily indicator/disease-state content turns conditional dietary advice into general rules without specifying target population, monitoring indicators, contraindications, or medical-care boundaries. The same logic applies to intermittent fasting, 16:8, and low-carbohydrate content in the method entry. The present study evaluates the structural position of these methods, with the risk position identified by the high proportion of rhythm schemes and templating within the entry.

This distinction helps avoid two extremes. Treating all concentration as harmful ignores the reasonable topical focus built into users’ search entries. Treating all concentration as user demand ignores how publicly visible content repeatedly shapes the meaning of healthy eating. This study therefore distinguishes focus from narrowing: entry focus is a normal match between user intent and content topic, whereas structural narrowing occurs when goal concentration further compresses method pathways, replaces food objects with products, strengthens templates, or weakens condition boundaries. Entry focus becomes meaningful as informational partiality only when these dimensions appear together.

### 5.3 Product entries: object substitution and boundary pressure

The product-substitution entry requires careful interpretation because it combines a high productized-object rate with the lowest Top3 structural-combination share. Product entries were diverse in goal-method combinations while strongly shifting the object of healthy eating toward meal replacements, protein powder, nutritional supplements, and meal-replacement foods. Narrowing in this entry therefore appears mainly at the level of what is taken to constitute healthy eating, rather than at the level of a single goal or pathway. The governance value lies in identifying when products are packaged as the primary solution to healthy eating, while allowing ordinary product-related discussion to remain distinguishable from higher-risk communication.

The relationship between productized objects and condition boundaries is especially important. The data show that the product entry had a high Q1 rate, a high rate of partial or missing conditions (A2 + A3) among Q1 posts, and insufficient coverage of detailed elements such as risks/contraindications, frequency, and duration. Supplement communication risks can arise from individual false claims, misleading constraints, communication structures, and influencer distortion (25, 26). When diet, fitness, and supplement content is commercially packaged, false health claims can gain communicative space more easily (51, 52), advertising formats can affect adolescents’ judgments of the credibility of fitness and supplement information (53), and perceived influencer credibility can enter intentions to purchase endorsed dietary supplements (54). This literature helps explain why product entries merit attention to commercial attributes, ordinary food alternatives, target populations, and risk warnings in governance.

Platform governance should avoid a one-size-fits-all approach here. Content related to meal replacements, protein powder, and supplements may include ordinary experience, product reviews, commercial promotion, health-food marketing, or health advice. Governance rules should therefore be stratified according to whether the content makes advice or effect claims, addresses special populations, substitutes for ordinary meals, or discloses commercial relationships. Commercial identification can refer to the Measures for the Administration of Internet Advertising (46), and product warnings can refer to the Guidelines for Warning Labels of Health Foods (47). Boundaries for health popularization should also refer to the negative behavior list for online health popularization by medical personnel (44) and regulations on medical popularization by self-media (45). The structural diagnosis proposed here can extend these regulations from single-item violation detection to entry monitoring: when a product entry persistently replaces basic foods with products while also lacking risk boundaries, the platform should increase prompts and review intensity.

### 5.4 High interaction as a monitoring cue

Interaction results are best read as visibility cues. Likes, comments, saves, and shares can describe the reach and engagement of food and nutrition information (30), and they can also serve as observational cues for the dissemination activity of health science misinformation (31), while content quality and sharing practices may be misaligned (32). In this study, highly interactive posts were more concentrated around weight, restriction-control, and templated structures, without higher rates of productized objects or condition insufficiency. High interaction therefore marks monitoring priority rather than a direct risk label.

High interaction provides a monitoring sequence. Weight, control, and templated content more readily receives public response, making these entries suitable front-end targets for routine monitoring. Platforms and professional health educators can first examine high-interaction content under fat-loss meals, low-calorie diets, fat-loss recipes, and weekly meal plans for absolutized promises, extreme restriction, missing substitution rules, or inapplicable situations. Low-interaction product and bodily indicator/disease-state entries also deserve boundary checks because their risk cues may appear through applicability conditions, risk warnings, and commercial attributes rather than through interaction popularity.

This distinction also answers the central question of the study. Under the evidence standard used here, namely repeated structures within publicly visible search-entry content, the sample shows entry-conditioned cues of informational partiality. Questions about platform-wide probability and algorithmic causation require ranking logs, exposure records, or user-level data. The high-interaction results show that some structures receive stronger public response, while product and indicator entries still require boundary checks even when interaction is lower.

### 5.5 Typological governance: from fact-checking to entry-level structural intervention

Fact-checking remains a foundation of healthy eating platform governance, and the results indicate a complementary role for entry-level structural intervention. For weight entries, fat-loss meal, low-calorie diet, and slimming-meal content can be supplemented with non-weight health goals, basic-food diversity, and prompts for long-term dietary sustainability. For bodily indicator/disease-state entries, glucose-control, low-GI, and uric-acid-lowering content can retain clearer disclosure of target population, monitoring conditions, risks or contraindications, and boundaries for medical consultation when advice or effect claims are made. Method entries can pair intermittent fasting, 16:8, carbohydrate cycling, and low-carbohydrate content with cycle length, stopping conditions, contraindicated populations, and alternatives, so that method rules are presented alongside their conditions of use.

Product entries require commercial identification, risk warnings, and ordinary-food alternatives to appear together. Platforms can set clearer content labels for meal replacements, protein powder, nutritional supplements, and meal-replacement food entries: experience sharing, ordinary food recommendation, commercial collaboration, health-food marketing, and professional advice should be distinguished. Once content claims weight loss, glucose control, indicator improvement, or meal substitution, it should prompt applicability limits and professional consultation. Template entries, meanwhile, should convert “replicable” into “adjustable.” Weekly meal plans, “eat as shown,” and diet check-in content should present portion ranges, ingredient substitutions, differences in energy needs, and inapplicable situations rather than only fixed templates.

Professional health education can extend beyond publishing more authoritative content. Short-form video media can carry information about improving diet quality (55), and social media can support healthy eating promotion (56, 57). Nutritionists and dietetic professionals have opportunities to enter platform communication, while also addressing ethical boundaries and practical constraints (58). Platform intervention tools further show that public health communication can embed nutrition decision support into digital environments (59). The present results suggest that professional health education can enter specific search entries directly: fat-loss entries can explain the relationship between weight goals and dietary quality; indicator entries can explain conditions of applicability for bodily indicator/disease-state diets; product entries can distinguish ordinary foods, health foods, and dietary supplements; and template entries can turn fixed meal plans into substitutable and adjustable dietary decision tools.

User-side governance can also address structural concentration in addition to truth and falsehood. Existing studies have examined false health information identification, online health persuasion strategies, social media health information credibility assessment, pseudo-health information correction, and distorted health information governance (60–64). These studies provide a foundation for understanding health information misleadingness, persuasion, and correction. The present results further show that risks in platform healthy eating content may also arise from entry-level structural concentration. Source credibility and health literacy influence public judgments of nutrition information (65, 66), and for young users, everyday health advice can create judgment difficulties across professionalism, credibility, and misinformation risk (67). Digital healthy eating literacy can therefore train users to seek authoritative sources and to identify entry bias, including entries that only discuss weight loss, center control pathways, present products as solutions, omit conditions of applicability, or write templates as rules for everyone to follow. Such literacy building can connect platform governance with user capability.

### 5.6 Limitations and future research

The main limitations concern sample coverage and the level of inference. The sample is not a probability sample of Xiaohongshu and cannot represent all healthy-eating content on the platform. The study instead used six semantic entry categories and 24 keywords to cover general healthy eating, weight management, bodily indicators, method mechanisms, product substitution, and templated arrangements. It collected 600 search-occurrence records and retained 594 unique core posts after deduplication. This design provides systematic coverage of entry semantics and supports comparison within the constructed corpus, but it does not support platform-wide prevalence estimates.

The medical accuracy of individual posts remains a task for professional fact-checking. This study instead diagnoses information structure. By separating advice or claim triggers from condition-disclosure status, the coding framework identifies which posts address readers with advice or effect claims and whether relevant conditions, warnings, or professional boundaries are disclosed. A2 (partial) and A3 (missing) therefore indicate incomplete applicability disclosure rather than verified medical error, legal violation, or health harm.

These limitations define the scope of generalization and causal interpretation. Within that scope, the descriptive finding remains that visible healthy-eating content differs across search entries and that some structures are more common among posts with stronger public response. Future work can add ranking logs, complete candidate-pool records, longitudinal observation, and user-level designs to examine temporal stability, actual exposure, and adoption.

## 6 Conclusion

Using a stratified sample of public Xiaohongshu image-text posts, this study developed and applied a diagnostic framework for structural narrowing in public digital health information. The pooled corpus was concentrated mainly in health goals and method pathways, while food objects retained some diversity. Search entries expressed different configurations: weight entries emphasized goals and control, indicator entries combined state management with applicability boundaries, method entries emphasized rules and templates, product entries foregrounded commercial objects and disclosure pressure, and templated entries emphasized replicable form. Highly interactive posts showed stronger weight, control, and templated structures; these associations are interpreted as public-response patterns, while recommendation, content quality, and user adoption require separate evidence. In this study, structural narrowing means the co-occurrence of several features within an entry, with the framework shifting attention from single-item truth or falsehood to entry-level structure and offering an upstream monitoring perspective for public digital-health information, preventive health governance, and health-data governance while remaining distinct from clinical, insurance, and patient-level research.

## Data Availability

The study used publicly visible Xiaohongshu image-text posts and did not retain author identities, user profiles, user IDs, note IDs, original links, or other traceable fields. The de-identified coding framework, variable definitions, paired-coding labels, Cohen’s kappa summary, confusion matrices, and analytical specifications are available from the corresponding author upon reasonable request. Search-ranking patterns, result-page numbers, page positions, and scrolling counts were not collected and are therefore not available.
